# Anastomotic leak after manual circular stapled left-sided bowel surgery: analysis of technology-, disease-, and patient-related factors

**DOI:** 10.1093/bjsopen/zrae089

**Published:** 2024-10-23

**Authors:** European Society of Coloproctology (ESCP) Circular Stapled Anastomosis Working Group and 2017 European Society of Coloproctology (ESCP) Collaborating Group, C Tong, N Jamous, N-D Schmitz, K Szwarcensztein, DG Morton, TD Pinkney, A El-Hussuna, N Battersby, A Bhangu, S Blackwell, N Buchs, S Chaudhri, D Dardanov, A Dulskas, A El-Hussuna, M Frasson, G Gallo, J Glasbey, J Keatley, M Kelly, C Knowles, YE Li, V McCourt, A Minaya-Bravo, P Neary, I Negoi, D Nepogodiev, F Pata, G Pellino, T Poskus, L Sanchez-Guillen, B Singh, E Sivrikoz, G van Ramshorst, O Zmora, TD Pinkney, R Perry, EL Magill, J Keatley, C Tong, SE Ahmed, M Abdalkoddus, A Abelevich, S Abraham, M Abraham-Nordling, SI Achkasov, M Adamina, C Agalar, F Agalar, T Agarwal, O Agcaoglu, F Agresta, G Ahmad, A Ainkov, R Aiupov, VS Aledo, A Aleksic, F Aleotti, D Alias, AS Allison, A Alonso, S Alonso, R Alós, Y Altinel, M Alvarez-Gallego, E Amorim, G Anania, PS Andreev, P Andrejevic, V Andriola, N Antonacci, F Antos, M Anwer, P Aonzo, JJ Arenal, B Arencibia, S Argeny, SJ Arnold, S Arolfo, DY Artioukh, MA Ashraf, MI Aslam, CR Asteria, M Atif, S Avital, M Bacchion, SM Bach, R Balestri, A Balfour, E Balik, I Baloyiannis, GS Banipal, JEM Baral, B Barišić, I Bartella, G Barugola, GA Bass, MR Bedford, A Bedzhanyan, A Belli, J Beltrán de Heredia, WA Bemelman, V Benčurik, A Benevento, DJ Bergkvist, JC Bernal-Sprekelsen, I Besznyák, V Bettencourt, AJ Beveridge, C Bhan, S Bilali, V Bilali, E Binboga, V Bintintan, A Birindelli, T Birsan, F Blanco-Antona, RLGM Blom, EG Boerma, M Bogdan, MZ Boland, P Bondeven, A Bondurri, J Broadhurst, SA Brown, P Buccianti, NC Buchs, P Buchwald, D Bugra, A Bursics, HLE Burton, CJ Buskens, C Bustamante Recuenco, C Cagigas-Fernandez, A Calero-Lillo, V Calu, I Camps, AE Canda, L Canning, S Cantafio, A Carpelan, MJ Carrillo Lopez, JM Carvas, M Carvello, J Castellvi, J Castillo, J Castillo-Diego, V Cavenaile, L Cayetano Paniagua, AA Ceccotti, J Cervera-Aldama, A Chabok, PC Chandrasinghe, N Chandratreya, SS Chaudhri, ZU Chaudhry, P Chirletti, J Chi-Yong Ngu, C Chouliaras, M Chowdhary, NA Chowdri, AB Christiano, P Christiansen, MA Citores, C Ciubotaru, C Ciuce, N Clemente, D Clerc, A Codina-Cazador, E Colak, L Colao García, D Coletta, F Colombo, TM Connelly, S Cornaglia, J Corte Real, J Costa Pereira, S Costa, E Cotte, ED Courtney, AP Coveney, P Crapa, DA Cristian, M Cuadrado, K Cuinas, MV Cuk, VV Cuk, MF Cunha, R Curinga, N Curtis, E Dainius, A d'Alessandro, RSJ Dalton, IR Daniels, D Dardanov, B Dauser, O Davydova, B De Andrés-Asenjo, EJR de Graaf, F De la Portilla, FB de Lacy, ECD De Laspra, B Defoort, T Dehli, L Del Prete, P Delrio, S Demirbas, A Demirkiran, FC Den Boer, S Di Saverio, A Diego, B Dieguez, M Diez-Alonso, I Dimitrijevic, B Dimitrios, N Dimitriou, G Dindelegan, S Dindyal, H Domingos, PG Doornebosch, S Dorot, M Draga, I Drami, A Dulskas, A Dzulkarnaen Zakaria, E Echazarreta-Gallego, Y Edden, M Egenvall, V Eismontas, A El Nakeeb, M El Sorogy, H Elfike, A Elgeidie, A El-Hussuna, M Elía Guedea, S Ellul, S El-Masry, U Elmore, SH Emile, O Enciu, JM Enriquez-Navascues, JC Epstein, D Escolà Ripoll, B Espina, E Espin-Basany, AM Estévez Diz, MD Evans, PA Farina, Fatayer, F Feliu, C Feo, CV Feo, J Fernando, F Feroci, L Ferreira, T Feryn, B Flor-Lorente, A Forero-Torres, N Francis, M Frasson, MR Freund, M Fróis Borges, A Frontali, AB Gallardo, R Galleano, G Gallo, D Garcia, LJ García Flórez, JA García Marín, J García Septiem, AM Garcia-Cabrera, JM García-González, E Garcia-Granero, M Garipov, R Gefen, P Gennadiy, S Gerkis, A Germain, S Germanos, L Gianotti, M Gil Santos, C Gingert, O Glehen, T Golda, M Gómez Ruiz, D Gonçalves, JS González, J Grainger, F Grama, C Grant, J Griniatsos, T Grolich, J Grosek, J Guevara-Martínez, B Gulcu, SK Gupta, SV Gurjar, S Haapaniemi, Y Hamad, M Hamid, J Hardt, RL Harries, GJC Harris, L Harsanyi, J Hayes, ER Hendriks, F Herbst, N Hermann, A Heuberger, R Hompes, A Hrora, M Hübner, H Huhtinen, L Hunt, M Hyöty, N Ibañez, D Ignjatovic, A Ilkanich, M Inama, MS Infantino, MR Iqbal, A Isik, O Isik, M Ismaiel, SO Ivanovich, V Jadhav, D Jajtner, V Jiménez Carneros, RM Jimenez-Rodriguez, V Jotautas, K Jukka, J Juloski, B Jung, Y Kara, U Karabacak, A Karachun, S Karagul, M Kassai, E Katorkin Sergei, D Katsaounis, IE Katsoulis, ME Kelly, B Kenjić, S Keogh-Bootland, D Khasan, A Khazov, SH Kho, GN Khrykov, AJ Kivelä, MD Kjaer, JS Knight, P Kocián, T Koëter, JLM Konsten, J Korček, D Korkolis, S Korsgen, IS Kostić, PM Krarup, P Krastev, I Krdzic, E Kreisler Moreno, Z Krivokapic, CJ Krones, D Kršul, N Kumar Kaul, F La Torre, N Lahodzich, CW Lai, JLB Laina, Z Lakkis, S Lamas, CP Lange, A Lauretta, KA Lee, J Lefèvre, T Lehtonen, CA Leo, KJ Leong, A Lepistö, L Licari, P Lizdenis, P Loftås, M Longhi, J Lopez-Dominguez, J López-Fernández, H Lovén, R Lozoya Trujillo, R Lunin, AP Luzzi, ML Lydrup, J Lykke, VM Maderuelo-Garcia, T Madsboell, AH Madsen, A Maffioli, MA Majbar, A Makhmudov, D Makhmudov, KI Malik, SS Malik, ZZ Mamedli, DK Manatakis, R Mankotia, J Maria, NM Mariani, K Marimuthu, F Marinello, F Marino, G Marom, N Maroni, I Maroulis, P Marsanic, HA Marsman, M Martí-Gallostra, ST Martin, J Martinez Alegre, A Martinez Manzano, R Martins, S Maslyankov, K McArdle, DR McArthur, C McFaul, D McWhirter, D Mege, A Mehraj, MZ Metwally, IH Metwally, M Millan, AS Miller, A Minaya-Bravo, A Mingoli, G Minguez Ruiz, C Minusa, B Mirshekar-Syahkal, M Mistrangelo, SS Mogoanta, I Mohamed, PH Möller, T Möller, M Molteni, S Mompart, B Monami, M Mondragon-Pritchard, Pedro Moniz-Pereira, D Montesdeoca Cabrera, M Morais, BJ Moran, G Moretto, M Morino, A Moscovici, S Muench, H Mukhtar, P Muller, A Muñoz-Duyos, A Muratore, P Muriel, P Myrelid, M Nachtergaele, H Nadav, K Nastos, A Navarro-Sánchez, I Negoi, A Nesbakken, G Nestler, J Nicholls, D Nicol, M Nikberg, JMS Nobre, J Nonner, G Norčič, S Norderval, MGA Norwood, J Nygren, JW O’Brien, PR O’Connell, J O'Kelly, N Okkabaz, M Oliveira-Cunha, GEEI Omar, P Onody, E Opocher, J Orhalmi, FJ Orts-Micó, GS Ozbalci, U Ozgen, BB Ozkan, E Ozturk, K Pace, MH Padín, SB Pandey, JA Pando, I Papaconstantinou, A Papadopoulos, G Papadopoulos, G Papp, S Paraskakis, Y Parc, P Parra Baños, FQ Parray, R Parvuletu, A Pascariello, I Pascual Migueláñez, F Pata, H Patel, PK Patel, HM Paterson, JC Patrón Uriburu, GC Pattacini, V Pavlov, A Pcolkins, EM Pellicer-Franco, E Peña Ros, HD Pérez, P Petkov, P Picarella, AJ Pikarsky, A Pisani Ceretti, E Platt, P Pletinckx, M Podda, D Popov, E Poskus, T Poskus, MC Prats, I Pravosudov, V Primo-Romaguera, V Prochazka, I Pros Ribas, D Proud, J Psaila, F Pullig, MS Qureshi Jinnah, J Rachadell Montero, D Radovanovic, Z Radovanovic, MM Rahman, R Rainho, N Rama, D Ramos, A Ramsanahie, A Rantala, A Rasulov, T Rautio, T Raymond, A Raza, A Reddy, B Refky, L Regusci, P Reissman, M Rems, ML Reyes-Diaz, R Riccardo, G Richiteanu, F Richter, A Rios, F Ris, FL Rodriguez, P Rodriguez Garcia, JA Rojo Lopez, M Romaniszyn, GM Romano, AS Romero, M Romero-Simó, A Roshan Lal, B Rossi, A Ruano Poblador, M Rubbini, I Rubio-Perez, H Ruiz, E Rullier, O Ryska, D Sabia, M Sacchi, N Saffaf, A Sakr, Z Saladzinskas, I Sales, M Salomon, S Salvans, NE Samalavicius, G Sammarco, GM Sampietro, D Samsonov, JL Sanchez-Garcia, L Sánchez-Guillén, E Sanchiz, G Šantak, J Santos Torres, F Saraceno, IS Sarici, PB Sarmah, G Savino, S Scabini, C Schafmayer, B Schiltz, A Schofield, R Scurtu, E Segalini, J Segelman, JJ Segura Sampedro, R Seicean, A Sekulic, D Selwyn, P Serrano Paz, J Shabbir, IA Shaikh, M Shalaby, A Sharma, A Shukla, N Shussman, ZA Siddiqui, P Siironen, P Sileri, P Silva-Vaz, JF Simoes, H Sinan, B Singh, A Sivins, G Skroubis, M Skrovina, AJ Skull, M Slavchev, M Slavin, AAP Slesser, CJ Smart, NJ Smart, K Smedh, S Smolarek, M Sokolov, O Sotona, D Spacca, A Spinelli, G Stanojevic, A Stearns, S Stefan, A Stift, J Stijns, V Stoyanov, D Straarup, R Strouhal, BM Stubbs, C Suero Rodríguez, U Sungurtekin, S Svagzdys, I Svastics, I Syk, MJM Tabares, A Tamelis, RG Tamhane, N Tamini, A Tamosiunas, SA Tan, PJ Tanis, SJ Tate, V Tercioti Junior, C Terzi, V Testa, MA Thaha, JC Tham, N Thavanesan, JE Theodore, C Tinoco, M Todorovic, A Tomazic, V Tomulescu, V Tonini, BR Toorenvliet, J Torkington, A Torrance, MJ Toscano, I Tóth, S Trampus, E Travaglio, I Trostchanky, N Truan, H Tulchinsky, V Turrado-Rodriguez, R Tutino, A Tzivanakis, GA Tzovaras, LW Unger, S Vaccari, CJ Vaizey, G Valero-Navarro, I Valverde Nuñez, K Van Belle, K Van Belle, I van den Berg, AAW van Geloven, YT Van Loon, L van Steensel, M Varcada, AV Vardanyan, P Varpe, VR Velchuru, J Vencius, D Venskutonis, M Vermaas, M Vertruyen, M Vicente-Ruiz, A Vignali, V Vigorita, M Vila Tura, D Vimalachandran, L Vincenti, L Viso, RGJ Visschers, YS Voronin, P Walega, WZ Wan Zainira, JH Wang, X Wang, R Wani, J Warusavitarne, A Warwick, N Wasserberg, DJ Weiss, E Westerduin, JR Wheat, I White, G Williams, GL Williams, TR Wilson, JM Wilson, D Winter, AM Wolthuis, MPK Wong, J Worsøe, E Xynos, S Yahia, T Yamamoto, A Yanishev, Z Zaidi, MA Zairul Azwan, S Zaman, A Zaránd, A Zarco, M Zawadzki, M Zelic, P Žeromskas, M Zilvetti, O Zmora

## Abstract

**Background:**

Anastomotic leak rates after colorectal surgery remain high. In most left-sided colon and rectal resection surgeries, a circular stapler is utilized to create the primary bowel anastomosis. However, it remains unclear whether a relationship between circular stapler technology and anastomotic leak in left-sided colorectal surgery exists.

**Methods:**

A post-hoc analysis was conducted using a prospectively collected data set of patients from the 2017 European Society of Coloproctology snapshot audit who underwent elective left-sided resection (left hemicolectomy, sigmoid colectomy, or rectal resection) with a manual circular stapled anastomosis. Rates of anastomotic leak and unplanned intensive care unit stay in association with manual circular stapling were assessed. Patient-, disease-, geographical-, and surgeon-related factors as well as stapler brand were explored using multivariable regression models to identify predictors of adverse outcomes.

**Results:**

Across 3305 procedures, 8.0% of patients had an anastomotic leak and 2.1% had an unplanned intensive care unit stay. Independent predictors of anastomotic leak were male sex, minimal-access surgery converted to open surgery, and anastomosis height C11 (lower third rectum) (all *P* < 0.050). Independent predictors of unplanned intensive care unit stay were minimal-access surgery converted to open surgery and American Society of Anesthesiologists grade IV (all *P* < 0.050). Stapler device brand was not a predictor of anastomotic leak or unplanned intensive care unit stay in multivariable regression analysis. There were no differences in rates of anastomotic leak and unplanned intensive care unit stay according to stapler head diameter, geographical region, or surgeon experience.

**Conclusion:**

In patients undergoing left-sided bowel anastomosis, choice of manual circular stapler, in terms of manufacturer or head diameter, is not associated with rates of anastomotic leak and unplanned intensive care unit stay.

## Teaser

Across 3305 prospectively collected left-sided colorectal procedures involving a circular stapled anastomosis, stapler device manufacturer was not found to be a predictor of anastomotic leak or unplanned ICU stay. There were also no differences in rates of anastomotic leak and unplanned ICU stay according to stapler head diameter, geographical region, or surgeon experience. Patient factors remain the strongest predictors of anastomotic leak. Modest modifications to stapler technology seem unlikely to generate significant patient benefit and more radical and transformational changes may need to be explored.

## Introduction

Up to one-third of patients experience some form of complication as a result of colorectal surgery, ranging from relatively minor (Clavien–Dindo grade I) to fatal (Clavien–Dindo grade V)^1^. These complications can have a negative long-term impact on patients’ quality of life^2,3^. Anastomotic leak (AL) and unplanned intensive care unit (ICU) stays are well-known complications of colorectal surgery^4,5^; AL is perhaps the most clinically significant complication^6,7^. AL can result in intra-abdominal abscess, wound infection, bowel obstruction, or rupture of the operation wound, or require reoperation^3,8^. Patients with AL consequently have a 14.5 times higher risk of multiple organ failure and a 23.7 times higher risk of sepsis, compared with patients without AL^8^, and mortality approaching 1 in 3 patients^5,9^. In addition to the clinical burden, AL and AL-associated reoperations or re-interventions place a high burden on patients^3,5^, who may suffer physically, psychologically, and in some cases financially due to the potential need for a stoma, the extended recovery interval, and missed work time^6^. ICU stays after colorectal surgery (with or without anastomosis) are also a costly complication^5^ and AL is consistently associated with greater ICU usage^10^. Furthermore, an unplanned ICU stay after colorectal surgery is typically attributable to post-operative morbidity^11^ and represents an independent risk factor for mortality^12^. Given the substantial burden of these surgical complications, prevention, early detection, and prompt treatment of AL, as well as the prevention or reduction of unplanned ICU stays, are essential to improve outcomes for patients.

There are a number of possible causes for a complication after anastomosis, including patient and procedural factors. Reported patient factors include malnutrition, obesity (BMI greater than 30 kg/m^2^), diabetes, and American Society of Anesthesiologists (ASA) grade greater than II^13–15^. Intraoperative factors include technical failure, such as improper use of a stapler, impaired bowel blood supply, and increased anastomotic tissue tension^16^. Circular staplers have been demonstrated to reduce the risk of AL *versus* handsewn sutures, particularly in the rectum and left colon; however, the use of a circular stapler does not eliminate this complication.

Male sex, low anterior resection, and patient co-morbidity are widely reported predictors of AL^15^. However, pre-surgery risk assessments for AL among patients undergoing colorectal surgery with anastomosis still lack predictive accuracy^17,18^. Reporting real-world outcomes after surgical intervention is consequently an important step in understanding the impact of these different risk factors. Since 2015, the European Society of Coloproctology (ESCP) has developed pioneering prospective cohort studies aimed at understanding real-world colorectal practice, addressing key research topics on right-sided resections in 2015 and left-sided resections in 2017^1,10,19–22^.

The objective of this analysis was to use the 2017 ESCP snapshot data to quantify rates of AL and unplanned ICU stay among patients undergoing a left hemicolectomy, sigmoid colectomy, or rectal resection for which the surgeon used a manual circular stapler. Another objective was to better understand the predictors associated with these complications by answering two research questions: is use of these staplers associated with AL and unplanned ICU stay?; and are any patient-, disease-, geographical-, surgeon-, or stapler-related factors predictors of these adverse outcomes?

## Methods

### Data collection

A post-hoc analysis was conducted using prospectively collected data from the 2017 ESCP international, observational snapshot audit of left colon, sigmoid, and rectal resections (as reported previously)^1,10,20–22^. Local investigators collected data for all eligible operations (left colon, sigmoid, and rectal resections) between February 2017 and June 2017 with follow-up through 30 days after surgery. Data were captured for 5641 patients at 335 sites across 49 countries, including 15 countries outside of Europe. Inclusion criteria were as per the ESCP protocol^1^, with the addition of age greater than or equal to 16 years and recorded use of manual circular stapled anastomosis in the primary procedure.

### Outcomes of interest

**Fig. 1:**
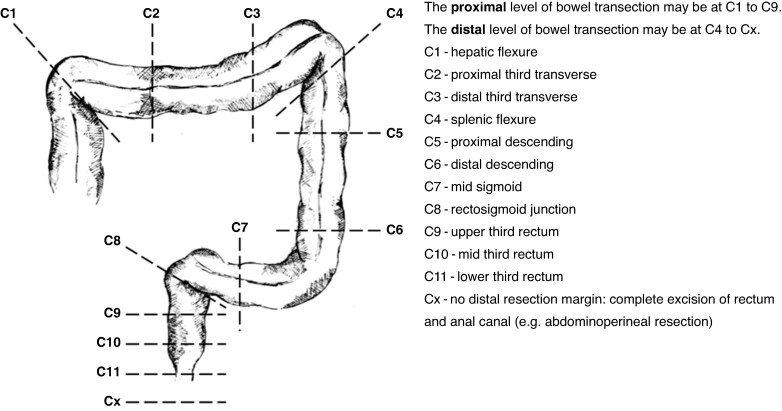
Definitions for height of resection margins (C1 through Cx) Adapted from ESCP Cohort Studies and Audits Committee^23^.

The primary outcomes of interest were to quantify the incidence of AL (defined as proven AL and/or intra-abdominal collection, as per the primary outcome measure in the parent ESCP study^23^) and unplanned ICU stay in patients undergoing a left hemicolectomy, sigmoid colectomy, or rectal resection for which the surgeon used a manual circular stapler, and to better understand predictors associated with complications after anastomosis. The schematic of operation resection margins used in the original ESCP snapshot audit is provided in *Fig. 1*.

Covariables used as potential predictors of circular stapling-related complications (AL and unplanned ICU stay) included patient demographics, clinical characteristics, and procedural characteristics, and are detailed in *Table S1*.

Finally, a sub-analysis was conducted in relation to the device used. For this purpose, device brands were anonymized and categorized as group A (all procedures using device brand 1 only), group B (procedures using a branded device excluding brand 1), or unrecorded (procedures using a device whose brand was not specified). A post-hoc exploratory analysis was also performed exploring the relationship between stapler head diameter and the same adverse outcomes.

### Statistical analyses

This was an exploratory, post-hoc analysis that aimed to generate hypotheses for both future research studies and for validation in further prospective ESCP snapshot audits and potential prospective controlled trials. No formal statistical hypothesis testing was conducted and all statistical tests and associated *P* values should be considered exploratory.

Data are presented as *n* (%) for categorical variables and as mean(s.d.) for continuous variables. A standardized mean difference (SMD) was calculated to measure the imbalance between two groups. A preference score analysis was conducted to confirm the comparability of the three anonymized brand groups^24^.

Multivariable analysis of complications by stapler brand (group A *versus* group B, excluding unrecorded) was conducted using a regularized logistic regression model (least absolute shrinkage and selection operator (LASSO) regression) to identify the variables associated with complications; all potential covariables at baseline (including stapler brand) were included and the final LASSO models deselected irrelevant covariables by shrinking their coefficients to zero. Excluding these covariables as complication predictors, OR estimates for binary outcomes are reported for all covariables with statistical significance (*P* < 0.050) from a logistic regression model for binary outcomes. Missing continuous variables (for age, body mass index (BMI), preoperative albumin, and preoperative haemoglobin) were imputed using the population median value. The unrecorded group was removed from the multivariable analysis because this group included a mixture of unknown brands and was not suitable for comparison with group A or group B.

Analysis was conducted using a chi-squared test for categorical variables and ANOVA for continuous variables. Subgroup analyses using descriptive statistics were conducted to summarize rates of AL and unplanned ICU stay for stapler head diameter, geographical region, and surgeon experience.

### Role of the funding source

The ESCP research network independently conducted data collection, subsequent data cleaning, and anonymization of the data set. Johnson & Johnson provided funding to ESCP to enable post-hoc, secondary analysis of the data set. This secondary analysis of the cleaned, anonymized data set was conducted by Johnson & Johnson, with clinical oversight and input from the ESCP. Manuscript development was funded by Johnson & Johnson.

## Results

### Patient population

**Fig. 2:**
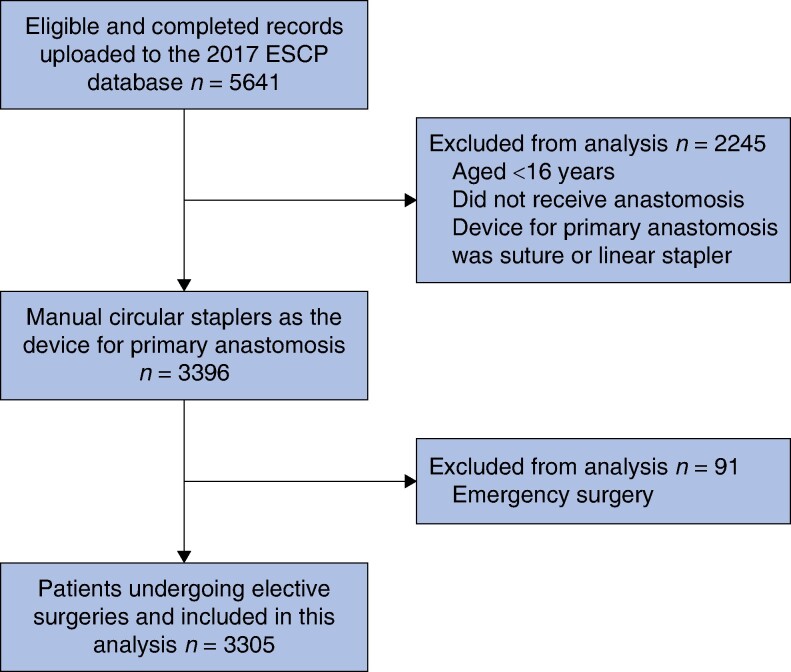
Patient flow chart for records included in the analysis ESCP, European Society of Coloproctology.

A total of 5641 eligible records were assessed for inclusion in the analysis, from which 3396 procedures using manual circular staplers were identified. After excluding emergency procedures, the analysis included 3305 procedures (*Fig. 2*). Overall, 8.0% of patients experienced AL and 2.1% of patients had an unplanned ICU stay. Patients were analysed within three defined groups: group A (1378 patients), group B (1738 patients), and unrecorded (189 patients).

Overall, the mean patient age was 64 years, 58% of patients were male, and the mean(s.d.) BMI was 26.8(4.7) kg/m^2^. Most patients were ASA grade II (55%) or grade III (27%), had no history of ischaemic heart disease/stroke (85.9%), were not diabetic (86.1%), had no history of anticoagulant treatment (87.2%), and had never smoked (59.6%) (*Table 1*).

**Table 1:** Patient demographics and clinical characteristics

	Group A (*n* = 1378)	Group B (*n* = 1738)	Unrecorded (*n* = 189)	Total (*n* = 3305)	P*
**Sex**					0.762
Male	58.5	57.4	59.3	57.9	
Female	41.5	42.6	40.7	42.1	
Age (years), mean(s.d.)	64.43(12.6)	64.1(12.3)	65.5(11.1)	64.3(12.3)	0.304
BMI (kg/m^2^), mean(s.d.)	26.9(4.9)	26.7(4.5)	26.3(4.8)	26.8(4.7)	0.189
**ASA grade**					
I	210 (15.2)	255 (14.7)	34 (18.0)	499 (15.1)	0.057
II	789 (57.3)	950 (54.7)	89 (47.1)	1828 (55.3)	
III	337 (24.5)	495 (28.5)	58 (30.7)	890 (26.9)	
IV	30 (2.2)	31 (1.8)	6 (3.2)	67 (2.0)	
Missing	12 (0.9)	7 (0.4)	2 (1.1)	21 (0.6)	
**History of anticoagulant treatment**					
Yes	154 (11.2)	236 (13.6)	30 (15.9)	420 (12.7)	0.055
No	1223 (88.8)	1501 (86.4)	159 (84.1)	2883 (87.2)	
Missing	1 (0.1)	1 (0.1)	0 (0.0)	2 (0.1)	
**History of diabetes mellitus**					
Yes, on any treatment	198 (14.4)	233 (13.4)	26 (13.8)	457 (13.8)	0.731
No	1177 (85.4)	1504 (86.5)	163 (86.2)	2844 (86.1)	
Missing	3 (0.2)	1 (0.1)	0 (0.0)	4 (0.1)	
**History of ischaemic heart disease/stroke**					
Yes	194 (14.1)	232 (13.3)	39 (20.6)	465 (14.1)	0.023
No	1182 (85.8)	1506 (86.7)	150 (79.4)	2838 (85.9)	
Missing	2 (0.1)	0 (0.0)	0 (0.0)	2 (0.1)	
**Smoking history**					
Current smoker	170 (12.3)	227 (13.1)	28 (14.8)	425 (12.9)	0.565
Never smoked	810 (58.8)	1049 (60.4)	111 (58.7)	1970 (59.6)	
Ex-smoker (quit >6 weeks ago)	365 (26.5)	410 (23.6)	46 (24.3)	821 (24.8)	
Ex-smoker (quit ≤6 weeks ago)	22 (1.6)	36 (2.1)	4 (2.1)	62 (1.9)	
Missing	11 (0.8)	16 (0.9)	0 (0.0)	27 (0.8)	

Values are *n* (%) unless otherwise indicated. *P-values are based on ANOVA for continuous variables, and chi-square for categorical variables. BMI, body mass index.

Baseline patient demographics and clinical characteristics were generally well matched across group A, group B, and the unrecorded group (*Table 1*). Only history of ischaemic heart disease/stroke, preoperative haemoglobin, preoperative enteric fistula, and preoperative intra-abdominal or pelvic abscess within 3 months of surgery had *P* < 0.050 and only preoperative haemoglobin had an SMD greater than 0.1 (*Table 1* and *Table S2*). The preference score analysis demonstrated that equipoise was observed in group A *versus* group B, but not group A *versus* the unrecorded group (*Fig. S1*).

### Predictors of anastomotic leak and unplanned ICU stay

**Fig. 3:**
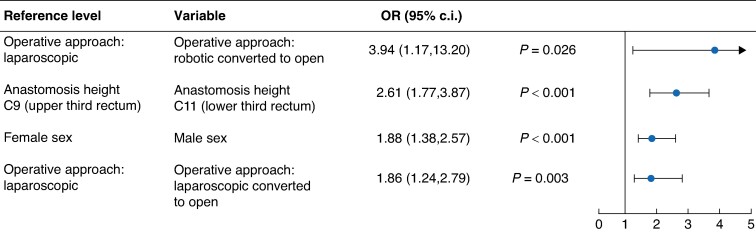
Predictors of anastomotic leak: group A *versus* group B only A rightward trend indicates a greater risk of complication *versus* the reference level and a leftward trend indicates a reduced risk of complication *versus* the reference level (that is OR values less than 1.0 indicate a reduced risk and OR values greater than 1.0 indicate an increased risk). Analysis was performed using regularized logistic regression models (least absolute shrinkage and selection operator; LASSO).

Male sex *versus* female sex (*P* < 0.001), laparoscopic converted to open operative approach *versus* laparoscopic operative approach (*P* = 0.003), robotic converted to open operative approach *versus* laparoscopic operative approach (*P* = 0.026), and anastomosis height C11 (lower third rectum) *versus* anastomosis height C9 (upper third rectum) (*P* < 0.001) were significant predictors of an increased risk of AL in multivariable analysis (*Fig. 3*).

**Fig. 4:**
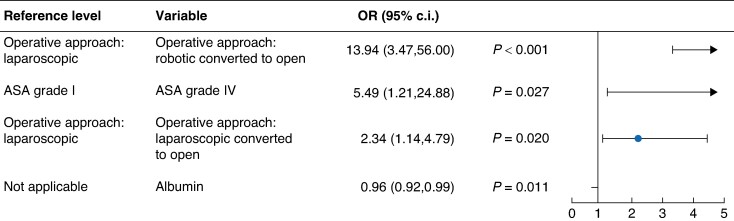
Predictors of unplanned ICU stay: group A *versus* group B only A rightward trend indicates a greater risk of complication *versus* the reference level and a leftward trend indicates a reduced risk of complication *versus* the reference level (that is OR values less than 1.0 indicate a reduced risk and OR values greater than 1.0 indicate an increased risk). There is no reference level required for albumin as it is a continuous variable. ICU, intensive care unit.

A total of three significant predictors of an increased risk of unplanned ICU stay were identified as follows: robotic converted to open operative approach *versus* laparoscopic operative approach (*P* < 0.001), laparoscopic converted to open operative approach *versus* laparoscopic operative approach (*P* = 0.020), and ASA grade IV *versus* ASA grade I (*P* = 0.027) (*Fig. 4*). Albumin was the only significant predictor of a reduced risk of unplanned ICU stay, predicting a 4% reduced risk with every one unit increase in albumin level (*P* = 0.011).

Stapler device brand was not identified as a significant predictor of AL (*Table S3*) or unplanned ICU stay in the multivariable regression models (*Figs 3*, *4*).

Exploratory descriptive analysis by stapler head diameter less than or equal to 29 mm (2213 patients; 66.8%) *versus* greater than 29 mm (1092 patients; 32.2%) showed no significant differences in the rates of AL and unplanned ICU stay according to stapler head diameter (*Table 2*). Similarly, analysis stratified by stapler head diameters of less than 28, 28, 29, 30/31, or greater than 31 mm found no differences in the rates of AL and unplanned ICU stay (*Table S4*).

**Table 2:** Incidence rates of complications stratified by stapler head diameter (pooled analysis, all procedures)

	≤29 mm (*n* = 2213)	>29 mm (*n* = 1092)	Total (*n* = 3305)	*P*
Anastomotic leak	168 (7.6)	96 (8.8)	264 (8.0)	0.231
Unplanned ICU stay	49 (2.2)	20 (1.8)	69 (2.1)	0.469

Values are *n* (%). Analysis used the chi-squared test. ICU, intensive care unit.

There was no difference in the rate of AL according to geographical region, with 4.4–9.2% of patients experiencing AL^3^ (*Table 3* and *Table S5*). There was a difference in the rate of unplanned ICU stay according to geographical region (*P* = 0.003) (*Table 3*); however, this difference should be interpreted with caution because of small numbers.

**Table 3:** Incidence rates of complications stratified by geographical region

	Non-EU (*n* = 228)	Eastern Europe (*n* = 369)	Northern Europe (*n* = 855)	Southern Europe (*n* = 1290)	Western Europe (*n* = 499)	Total (*n* = 3241)	*P*
Anastomotic leak	10 (4.4)	32 (8.7)	79 (9.2)	97 (7.5)	40 (8.0)	258 (8.0)	0.166
Unplanned ICU stay	5 (2.2)	0 (0.0)	25 (2.9)	21 (1.6)	17 (3.4)	68 (2.1)	0.003

Values are *n* (%). Analysis used the chi-squared test. The null hypothesis was that there was no difference among the five groups. EU, European Union; ICU, intensive care unit.

There were no differences in incidence rates according to surgeon experience, with similar rates of AL and unplanned ICU stay for trainee and consultant surgeons (*Table 4* and *Table S6*).

**Table 4:** Incidence rates of complications stratified by surgeon in charge

	Trainee (*n* = 253)	Consultant (*n* = 3049)	Missing (*n* = 3)	Total (*n* = 3305)	*P*
Anastomotic leak	18 (7.1)	246 (8.1)	0 (0.0)	264 (8.0)	0.760
Unplanned ICU stay	5 (2.0)	64 (2.1)	0 (0.0)	69 (2.1)	0.960

Values are *n* (%). Analysis used the chi-squared test. The null hypothesis was that there was no difference among the three groups. ICU, intensive care unit.

### Use of manual circular stapler technology and anastomotic leak or unplanned ICU stay

The rate of AL was comparable across the three device groups (*Table 5*) and bivariable analysis did not identify any significant effects of stapler device brand on rates of AL and unplanned ICU stay.

**Table 5:** Incidence rates of complications stratified by stapler brand (bivariable analysis)

	Group A (*n* = 1378)	Group B (*n* = 1738)	Unrecorded (*n* = 189)	Total (*n* = 3305)	*P*
Anastomotic leak	114 (8.3)	137 (7.9)	13 (6.9)	264 (8.0)	0.781
Unplanned ICU stay	34 (2.5)	31 (1.8)	4 (2.1)	69 (2.1)	0.415

Values are *n* (%). Analysis used the chi-squared test. The null hypothesis was that there was no difference among the three groups. ICU, intensive care unit.

## Discussion

Complications after colorectal surgery are associated with worse patient outcomes^2,16,25^. Despite advances in surgical technology and techniques, AL in colorectal surgery continues to occur at unacceptable rates, causing pelvic sepsis, risk of permanent stoma, and increased all-cause mortality^16^. Unplanned ICU stay after colorectal surgery is a costly complication^12^, also associated with increased mortality^11^. This study explores the relationship between a specific technology (the manual circular stapling device) and the occurrence of AL/unplanned ICU stay in an international cohort of patients. Further analyses sought to determine whether there were differences in rates of AL and unplanned ICU stay according to stapler brand and head diameter, according to stapler use by geographical region, and according to surgeon experience regarding stapler use.

Before conducting this analysis, a feasibility assessment confirmed that baseline patient demographics and clinical characteristics were generally well matched across the three stapler groups, although the unrecorded group was a small and potentially heterogeneous group. There was no significant effect of stapler device brand on rates of AL and unplanned ICU stay, with similar rates across device brands. However, it is possible that brand selection between patient groups may vary between regions and surgeons, and these interactions were not examined in this study.

Patient demographics and clinical characteristics were significant predictors of AL and unplanned ICU stay, consistent with the authors’ previous report on left-sided anastomosis not limited to manual circular staples^10^, suggesting these patient factors are not influenced by the use of manual circular stapling.

The rate of AL in the present study (8%) is consistent with the range previously reported in the literature (4–15%)^3,13,15,26^. Multivariable analysis identified male sex *versus* female sex, minimal-access surgery converted to open surgery *versus* laparoscopic surgery, and anastomosis height C11 (lower third rectum) *versus* anastomosis height C9 (upper third rectum) as key predictors of AL, consistent with the literature^13,15^. Notably, procedures that required conversion to open surgery resulted in a two to four times greater risk of AL, compared with those completed via minimally invasive approaches. However, patients requiring conversion to open surgery may represent those who were high risk before surgery and the rate of complications is typically higher for open surgery^25^; some complications in the conversion to open surgery group may therefore be due to the open surgery itself.

Robotic or laparoscopic procedures requiring conversion to open surgery *versus* laparoscopic approach, as well as ASA grade IV *versus* ASA grade I, were significant predictors of an increased risk of unplanned ICU stay. In previous studies, preoperative surgical judgement of risk of AL was reported to have low accuracy for prediction of AL^17,18^, thus improving patient selection processes for surgery may help reduce complication rates. There was a significant association between albumin level and a reduced risk of unplanned ICU stay, with every one unit increase in albumin inferring a 4% reduced risk. This is consistent with previous literature describing low preoperative albumin level as an independent predictor of increased morbidity after colorectal surgery^7^. The patient demographics and clinical characteristics identified as predictors of an increased risk of AL in the present study may aid surgeon decision-making for patients undergoing left hemicolectomy, sigmoid colectomy, or rectal resection operations requiring anastomosis.

Finally, there was no association between the incidence of AL or unplanned ICU stay and stapler head diameter, geographical region, or surgeon experience, apart from a difference in the incidence of unplanned ICU stay according to geographical region. As these were descriptive analyses, these factors were not included in the multivariable models; however, the difference observed in the incidence of unplanned ICU stay indicates that future research on these factors may be valuable.

The present analysis was a post-hoc analysis, based on non-randomized, prospectively collected data, and all results should be interpreted in consideration of the limitations inherent to this study design. The patient demographic and clinical factors available, including co-morbidities, were limited to those collected according to the ESCP audit protocol; consequently, analysis of other patient-related predictors of AL was not possible. Furthermore, the data were predominantly collected in Europe and were limited to those hospitals that participated in the ESCP audit, potentially limiting the generalizability of the results. It should also be acknowledged that a complex series of factors influence the availability of stapling devices at each hospital, including supply chains and procurement decisions often made at a non-clinical level. This study did not include any assessment of health economic or cost-effectiveness profiles between device brands. It is also true that, in some resource-limited settings, the availability of circular stapling devices may be very limited. In such circumstances a handsewn anastomosis may be utilized by some surgeons. This post-hoc analysis only included patients undergoing circular stapled anastomosis; results from the entire cohort including those having a handsewn, linear stapled, or no anastomosis have been reported previously^1,10,21,22^.

As the fundamental aim of this research was to inform future research questions about complications after anastomosis, no formal statistical hypothesis testing was conducted. Subgroup analyses for stapler head diameter, geographical region, and surgeon experience are descriptive and all statistical tests and *P* values for statistical significance should be considered exploratory.

Overall, no link between AL and manual circular stapler device was documented. The descriptive analysis also showed no differences in the rate of AL according to stapler head diameter, geographical region, or surgeon experience. If surgeon training or device features are a cause of AL, these appear to have a similar effect across device brands, geographical regions, and surgeon experience, while patient factors remain the strongest predictors of AL.

## Collaborators

**European Society of Coloproctology (ESCP) Circular Stapled Anastomosis Working Group and 2017 European Society of Coloproctology (ESCP) Collaborating Group**

Study design, interpretation and writing group: Tong C, Jamous N, Schmitz N-D, Szwarcensztein K, Morton DG, Pinkney TD (chair). Central Team: El-Hussuna A (2017 ESCP Audit Lead), Battersby N, Bhangu A, Blackwell S, Buchs N, Chaudhri S, Dardanov D, Dulskas A, El-Hussuna A, Frasson M, Gallo G, Glasbey J, Keatley J, Kelly M, Knowles C, Li YE, McCourt V, Minaya-Bravo A, Neary P, Negoi I, Nepogodiev D, Pata F, Pellino G, Poskus T, Sanchez-Guillen L, Singh B, Sivrikoz E, van Ramshorst G, Zmora O, Pinkney TD, Perry R, Magill EL, Keatley J, Tong C. Site-level collaborators: Ahmed SE, Abdalkoddus M, Abelevich A, Abraham S, Abraham-Nordling M, Achkasov SI, Adamina M, Agalar C, Agalar F, Agarwal T, Agcaoglu O, Agresta F, Ahmad G, Ainkov A, Aiupov R, Aledo VS, Aleksic A, Aleotti F, Alias D, Allison AS, Alonso A, Alonso S, Alós R, Altinel Y, Alvarez-Gallego M, Amorim E, Anania G, Andreev PS, Andrejevic P, Andriola V, Antonacci N, Antos F, Anwer M, Aonzo P, Arenal JJ, Arencibia B, Argeny S, Arnold SJ, Arolfo S, Artioukh DY, Ashraf MA, Aslam MI, Asteria CR, Atif M, Avital S, Bacchion M, Bach SM, Balestri R, Balfour A, Balik E, Baloyiannis I, Banipal GS, Baral JEM, Barišić B, Bartella I, Barugola G, Bass GA, Bedford MR, Bedzhanyan A, Belli A, Beltrán de Heredia J, Bemelman WA, Benčurik V, Benevento A, Bergkvist DJ, Bernal-Sprekelsen JC, Besznyák I, Bettencourt V, Beveridge AJ, Bhan C, Bilali S, Bilali V, Binboga E, Bintintan V, Birindelli A, Birsan T, Blanco-Antona F, Blom RLGM, Boerma EG, Bogdan M, Boland MZ, Bondeven P, Bondurri A, Broadhurst J, Brown SA, Buccianti P, Buchs NC, Buchwald P, Bugra D, Bursics A, Burton HLE, Buskens CJ, Bustamante Recuenco C, Cagigas-Fernandez C, Calero-Lillo A, Calu V, Camps I, Canda AE, Canning L, Cantafio S, Carpelan A, Carrillo Lopez MJ, Carvas JM, Carvello M, Castellvi J, Castillo J, Castillo-Diego J, Cavenaile V, Cayetano Paniagua L, Ceccotti AA, Cervera-Aldama J, Chabok A, Chandrasinghe PC, Chandratreya N, Chaudhri SS, Chaudhry ZU, Chirletti P, Chi-Yong Ngu J, Chouliaras C, Chowdhary M, Chowdri NA, Christiano AB, Christiansen P, Citores MA, Ciubotaru C, Ciuce C, Clemente N, Clerc D, Codina-Cazador A, Colak E, Colao García L, Coletta D, Colombo F, Connelly TM, Cornaglia S, Corte Real J, Costa Pereira J, Costa S, Cotte E, Courtney ED, Coveney AP, Crapa P, Cristian DA, Cuadrado M, Cuinas K, Cuk MV, Cuk VV, Cunha MF, Curinga R, Curtis N, Dainius E, d'Alessandro A, Dalton RSJ, Daniels IR, Dardanov D, Dauser B, Davydova O, De Andrés-Asenjo B, de Graaf EJR, De la Portilla F, de Lacy FB, De Laspra ECD, Defoort B, Dehli T, Del Prete L, Delrio P, Demirbas S, Demirkiran A, Den Boer FC, Di Saverio S, Diego A, Dieguez B, Diez-Alonso M, Dimitrijevic I, Dimitrios B, Dimitriou N, Dindelegan G, Dindyal S, Domingos H, Doornebosch PG, Dorot S, Draga M, Drami I, Dulskas A, Dzulkarnaen Zakaria A, Echazarreta-Gallego E, Edden Y, Egenvall M, Eismontas V, El Nakeeb A, El Sorogy M, Elfike H, Elgeidie A, El-Hussuna A, Elía Guedea M, Ellul S, El-Masry S, Elmore U, Emile SH, Enciu O, Enriquez-Navascues JM, Epstein JC, Escolà Ripoll D, Espina B, Espin-Basany E, Estévez Diz AM, Evans MD, Farina PA, Fatayer, Feliu F, Feo C, Feo CV, Fernando J, Feroci F, Ferreira L, Feryn T, Flor-Lorente B, Forero-Torres A, Francis N, Frasson M, Freund MR, Fróis Borges M, Frontali A, Gallardo AB, Galleano R, Gallo G, Garcia D, García Flórez LJ, García Marín JA, García Septiem J, Garcia-Cabrera AM, García-González JM, Garcia-Granero E, Garipov M, Gefen R, Gennadiy P, Gerkis S, Germain A, Germanos S, Gianotti L, Gil Santos M, Gingert C, Glehen O, Golda T, Gómez Ruiz M, Gonçalves D, González, JS, Grainger J, Grama F, Grant C, Griniatsos J, Grolich T, Grosek J, Guevara-Martínez J, Gulcu B, Gupta, SK, Gurjar SV, Haapaniemi S, Hamad Y, Hamid M, Hardt J, Harries RL, Harris GJC, Harsanyi L, Hayes J, Hendriks ER, Herbst F, Hermann N, Heuberger A, Hompes R, Hrora A, Hübner M, Huhtinen H, Hunt L, Hyöty M, Ibañez N, Ignjatovic D, Ilkanich A, Inama M, Infantino MS, Iqbal MR, Isik A, Isik O, Ismaiel M, Ivanovich SO, Jadhav V, Jajtner D, Jiménez Carneros V, Jimenez-Rodriguez RM, Jotautas V, Jukka K, Juloski J, Jung B, Kara Y, Karabacak U, Karachun A, Karagul S, Kassai M, Katorkin Sergei E, Katsaounis D, Katsoulis IE, Kelly ME, Kenjić B, Keogh-Bootland S, Khasan D, Khazov A, Kho SH, Khrykov GN, Kivelä AJ, Kjaer MD, Knight JS, Kocián P, Koëter T, Konsten JLM, Korček J, Korkolis D, Korsgen S, Kostić IS, Krarup PM, Krastev P, Krdzic I, Kreisler Moreno E, Krivokapic Z, Krones CJ, Kršul D, Kumar Kaul N, La Torre F, Lahodzich N, Lai CW, Laina JLB, Lakkis Z, Lamas S, Lange CP, Lauretta A, Lee KA, Lefèvre J, Lehtonen T, Leo CA, Leong KJ, Lepistö A, Licari L, Lizdenis P, Loftås P, Longhi M, Lopez-Dominguez J, López-Fernández J, Lovén H, Lozoya Trujillo R, Lunin R, Luzzi AP, Lydrup ML, Lykke J, Maderuelo-Garcia VM, Madsboell T, Madsen AH, Maffioli A, Majbar MA, Makhmudov A, Makhmudov D, Malik KI, Malik SS, Mamedli ZZ, Manatakis DK, Mankotia R, Maria J, Mariani NM, Marimuthu K, Marinello F, Marino F, Marom G, Maroni N, Maroulis I, Marsanic P, Marsman HA, Martí-Gallostra M, Martin ST, Martinez Alegre J, Martinez Manzano A, Martins R, Maslyankov S, McArdle K, McArthur DR, McFaul C, McWhirter D, Mege D, Mehraj A, Metwally MZ, Metwally IH, Millan M, Miller AS, Minaya-Bravo A, Mingoli A, Minguez Ruiz G, Minusa C, Mirshekar-Syahkal B, Mistrangelo M, Mogoanta SS, Mohamed I, Möller PH, Möller T, Molteni M, Mompart S, Monami B, Mondragon-Pritchard M, Moniz-Pereira Pedro, Montesdeoca Cabrera D, Morais M, Moran BJ, Moretto G, Morino M, Moscovici A, Muench S, Mukhtar H, Muller P, Muñoz-Duyos A, Muratore A, Muriel P, Myrelid P, Nachtergaele M, Nadav H, Nastos K, Navarro-Sánchez A, Negoi I, Nesbakken A, Nestler G, Nicholls J, Nicol D, Nikberg M, Nobre JMS, Nonner J, Norčič G, Norderval S, Norwood MGA, Nygren J, O’Brien JW, O’Connell PR, O'Kelly J, Okkabaz N, Oliveira-Cunha M, Omar GEEI, Onody P, Opocher E, Orhalmi J, Orts-Micó FJ, Ozbalci GS, Ozgen U, Ozkan BB, Ozturk E, Pace K, Padín MH, Pandey SB, Pando JA, Papaconstantinou I, Papadopoulos A, Papadopoulos G, Papp G, Paraskakis S, Parc Y, Parra Baños P, Parray FQ, Parvuletu R, Pascariello A, Pascual Migueláñez I, Pata F, Patel H, Patel PK, Paterson HM, Patrón Uriburu JC, Pattacini GC, Pavlov V, Pcolkins A, Pellicer-Franco EM, Peña Ros E, Pérez HD, Petkov P, Picarella P, Pikarsky AJ, Pisani Ceretti A, Platt E, Pletinckx P, Podda M, Popov D, Poskus E, Poskus T, Prats MC, Pravosudov I, Primo-Romaguera V, Prochazka V, Pros Ribas I, Proud D, Psaila J, Pullig F, Qureshi Jinnah MS, Rachadell Montero J, Radovanovic D, Radovanovic Z, Rahman MM, Rainho R, Rama N, Ramos D, Ramsanahie, A, Rantala A, Rasulov A, Rautio T, Raymond T, Raza A, Reddy A, Refky B, Regusci L, Reissman P, Rems M, Reyes-Diaz ML, Riccardo R, Richiteanu G, Richter F, Rios A, Ris F, Rodriguez FL, Rodriguez Garcia P, Rojo Lopez JA, Romaniszyn M, Romano GM, Romero AS, Romero-Simó M, Roshan Lal A, Rossi B, Ruano Poblador A, Rubbini M, Rubio-Perez I, Ruiz H, Rullier E, Ryska O, Sabia D, Sacchi M, Saffaf N, Sakr A, Saladzinskas Z, Sales I, Salomon M, Salvans S, Samalavicius NE, Sammarco G, Sampietro GM, Samsonov D, Sanchez-Garcia JL, Sánchez-Guillén L, Sanchiz E, Šantak G, Santos Torres J, Saraceno F, Sarici IS, Sarmah PB, Savino G, Scabini S, Schafmayer C, Schiltz B, Schofield A, Scurtu R, Segalini E, Segelman J, Segura Sampedro JJ, Seicean R, Sekulic A, Selwyn D, Serrano Paz P, Shabbir J, Shaikh IA, Shalaby M, Sharma A, Shukla A, Shussman N, Siddiqui ZA, Siironen P, Sileri P, Silva-Vaz P, Simoes JF, Sinan H, Singh B, Sivins A, Skroubis G, Skrovina M, Skull AJ, Slavchev M, Slavin M, Slesser AAP, Smart CJ, Smart NJ, Smedh K, Smolarek S, Sokolov M, Sotona O, Spacca D, Spinelli A, Stanojevic G, Stearns A, Stefan S, Stift A, Stijns J, Stoyanov V, Straarup D, Strouhal R, Stubbs BM, Suero Rodríguez C, Sungurtekin U, Svagzdys S, Svastics I, Syk I, Tabares MJM, Tamelis A, Tamhane RG, Tamini N, Tamosiunas A, Tan SA, Tanis PJ, Tate SJ, Tercioti Junior V, Terzi C, Testa V, Thaha MA, Tham JC, Thavanesan N, Theodore JE, Tinoco C, Todorovic M, Tomazic A, Tomulescu V, Tonini V, Toorenvliet BR, Torkington J, Torrance A, Toscano MJ, Tóth I, Trampus S, Travaglio E, Trostchanky I, Truan N, Tulchinsky H, Turrado-Rodriguez V, Tutino R, Tzivanakis A, Tzovaras GA, Unger LW, Vaccari S, Vaizey CJ, Valero-Navarro G, Valverde Nuñez I, Van Belle K, Van Belle K, van den Berg I, van Geloven AAW, Van Loon YT, van Steensel L, Varcada M, Vardanyan AV, Varpe P, Velchuru VR, Vencius J, Venskutonis D, Vermaas M, Vertruyen M, Vicente-Ruiz M, Vignali A, Vigorita V, Vila Tura M, Vimalachandran D, Vincenti L, Viso L, Visschers RGJ, Voronin YS, Walega P, Wan Zainira WZ, Wang JH, Wang X, Wani R, Warusavitarne J, Warwick A, Wasserberg N, Weiss DJ, Westerduin E, Wheat JR, White I, Williams G, Williams GL, Wilson TR, Wilson JM, Winter D, Wolthuis AM, Wong MPK, Worsøe J, Xynos E, Yahia S, Yamamoto T, Yanishev A, Zaidi Z, Zairul Azwan MA, Zaman S, Zaránd A, Zarco A, Zawadzki M, Zelic M, Žeromskas P, Zilvetti M, Zmora O

## Supplementary Material

zrae089_Supplementary_Data

## Data Availability

Data sharing requests will be considered by the management group upon written request to the corresponding author. Deidentified participant data or other pre-specified data will be available subject to a written proposal and a signed data sharing agreement.
