# Supplementary material for: Anastomotic leak after manual circular stapled left-sided bowel surgery: analysis of technology-, disease-, and patient-related factors
Source: BJS Open. 2024 Oct 23;8(5):zrae089. doi: 10.1093/bjsopen/zrae089 (PMC11498054; doi:10.1093/bjsopen/zrae089)
Supplement: zrae089_Supplementary_Data [file zrae089_supplementary_data.docx]

**Anastomotic leak after manual circular stapled left sided bowel surgery – an analysis of technology-, disease-, and patient-related factors**

The European Society of Coloproctology (ESCP) Circular Stapled Anastomosis Working Group^1^ and the 2017 European Society of Coloproctology (ESCP) Collaborating Group^1^

^1^European Society of Coloproctology (ESCP) Cohort Studies Committee, Academic Department of Surgery, University of Birmingham, Birmingham, UK

**Corresponding author:**

Professor Thomas D Pinkney, MBChB, MD, MMedEd, FRCS

Academic Department of Surgery, University of Birmingham

Birmingham, B15 2TH, UK

Email: thomas.pinkney@uhb.nhs.uk

Phone: +44 121 371 5888

<https://orcid.org/0000-0001-7320-6673>

| **Supplementary Materials - Index** | Page |
| --- | --- |
| Table S1 | *page 2* |
| Table S2 | *page 3* |
| Figure S1 | *Page 4* |
| Table S3 | *page 6* |
| Table S4 | *page 7* |
| Table S5 | *page 8* |
| Table S6 | *page 9* |
| Detailed list of collaborators and contributions | *page 9* |

**Supplementary Tables**

Table S1. Covariates used as potential predictors for circular stapling-related complications

| **Patient demographics** | **Clinical characteristics:** | **Procedural characteristics** |
| --- | --- | --- |
| - Age - Sex - Asa score - History of ischaemic heart disease or stroke - Anticoagulant treatment - Diabetes - Smoking history - Body mass index | - Post op histology (benign polyp, cancer, Crohn’s disease, diverticular disease, ulcerative colitis) - Other (pre-op albumin, haemoglobin, pre-op enteric fistula, abscess [yes/no]) | - Region or cluster* - Stapler brand - Admission type (elective, expedited ≤2 weeks) - Surgical approach (open, laparoscopic, robotic assisted) - Pre-operative bowel preparation - Nutritional support (oral, parenteral, enteral) - Surgeon in charge (Trainee, Consultant) - Level of bowel transection - Anastomosis configuration (side to side, side to end, end to end) - Defunctioning stoma (Yes vs No) |

* defined as Northern Europe, Eastern Europe, Southern Europe, Western Europe or non-EU

Table S2. Additional patient demographics and clinical characteristics

|  | **Group A (N=1,378)** | **Group B (N=1,738)** | **Unrecorded (N=189)** | **Total (N=3,305)** | **P-value**** |
| --- | --- | --- | --- | --- | --- |
| Urgency of surgery, n (%) | | | | |  |
| Elective | 1,231 (89.3%) | 1,578 (90.8%) | 166 (87.8%) | 2,975 (90.0%) | 0.236 |
| Expedited (within 2 weeks) | 147 (10.7%) | 160 (9.2%) | 23 (12.2%) | 330 (10.0%) |  |
| Pre-operative albumin | | | | |  |
| Mean (SD) | 39.5 (7.2) | 39.6 (6.3) | 39.8 (6.2) | 39.5 (6.7) | 0.845 |
| Missing, n (%) | 227 (16.5%) | 288 (16.6%) | 36 (19.0%) | 551 (16.7%) |  |
| Pre-operative haemoglobin* | | | | |  |
| Mean (SD), g/dl | 12.9 (2.2) | 12.7 (2.3) | 13.0 (1.9) | 12.8 (2.2) | 0.022 |
| Missing, n | 11 | 17 | 3 | 31 |  |
| Pre-operative enteric fistula, n (%) |  |  |  |  |  |
| Yes | 65 (4.7%) | 43 (2.5%) | 4 (2.1%) | 112 (3.4%) | 0.002 |
| No | 1,311 (95.1%) | 1,695 (97.5%) | 185 (97.9%) | 3,191 (96.6%) |  |
| Missing | 2 (0.1%) | 0 (0.0%) | 0 (0.0%) | 2 (0.1%) |  |
| Pre-operative intra-abdominal or pelvic abscess, within 3 months of surgery, n (%) | | | | |  |
| Yes | 66 (4.8%) | 47 (2.7%) | 5 (2.6%) | 118 (3.6%) | 0.006 |
| No | 1,310 (95.1%) | 1,691 (97.3%) | 184 (97.4%) | 3,185 (96.4%) |  |
| Missing | 2 (0.1%) | 0 (0.0%) | 0 (0.0%) | 2 (0.1%) |  |
| Height of anastomosis | | | | |  |
| C4 (splenic flexure) to C8 (rectosigmoid junction) | 363 (26.4%) | 542 (31.2%) | 56 (29.6%) | 961 (29.1%) | 0.059 |
| C9 (upper third rectum) | 391 (28.4%) | 474 (27.3%) | 46 (24.3%) | 911 (27.6%) |  |
| C10 (mid third rectum) | 298 (21.7%) | 368 (21.2%) | 48 (25.4%) | 714 (21.6%) |  |
| C11 (third lower rectum) | 318 (23.1%) | 348 (20.0%) | 39 (20.6%) | 705 (21.3%) |  |
| Other | 6 (0..4%) | 6 (0.3%) | 0 (0.0%) | 12 (0.4%) |  |
| Missing (not answered) | 2 (0.1%) | 0 (0.0%) | 0 (0.0%) | 2 (0.1%) |  |

Abbreviations: ASA, American Society of Anaesthesiologists Classification; BMI, body mass index; dl, decilitre; IHD, ischaemic heart disease; SD, standard deviations.
Data are presented as mean (SD) unless otherwise stated.
* Standardised mean difference >0.1; analysed for Group A vs. Group B excluding Unrecorded.

** chi-square test for categorical variables and one way ANOVA for continuous variables

Figure S1. Preference Score Distributions of the three anonymised brand groups of stapler

Empirical equipoise is accepted if at least half of the distribution of each group are to patients with a preference score of between 0.3 and 0.7.


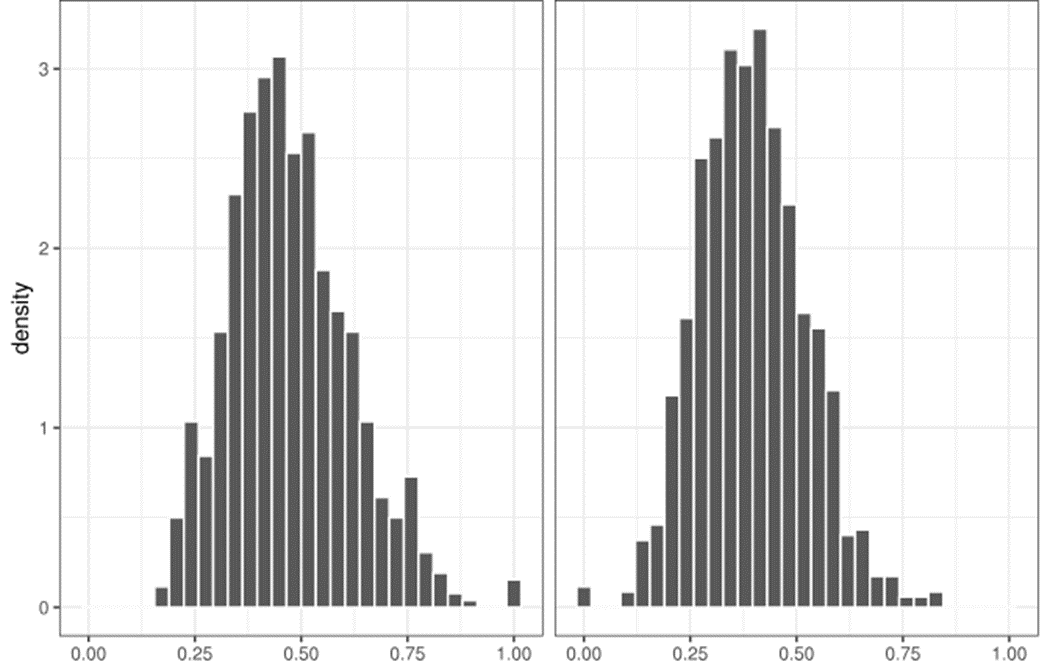


Group A Group B

Probability of getting Group A

84.0% of patients in Group A and 84.6% in Group B with preference score between 0.3 and 0.7, thus empirical equipoise is established.


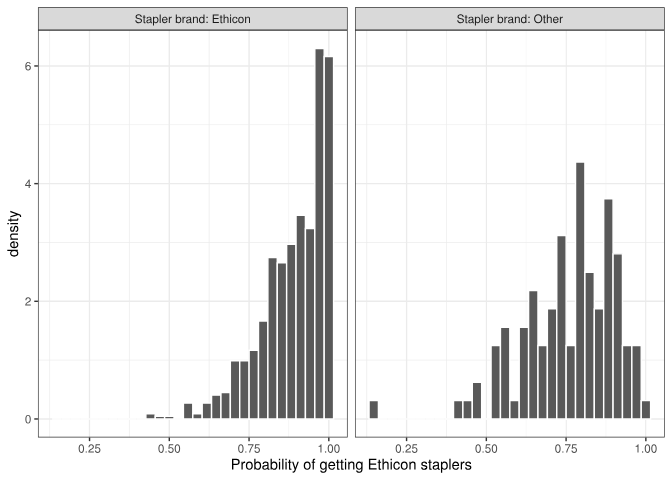


Group A Unrecorded

Probability of getting Group A

47.5% of patients in Group A and 51.4% in Group B with preference score between 0.3 and 0.7, thus empirical equipoise is not established.

Table S3. Rate of complications rates by stapler brand (bivariate analysis)

|  | **Group A**  **(N=1,378)** | **Group B**  **(N=1,738)** | **Unrecorded**  **(N=189)** | **Total**  **(N=3,305)** | **P value** |
| --- | --- | --- | --- | --- | --- |
| Proven leak and/or collection | 114 (8.3%) | 137 (7.9%) | 13 (6.9%) | 264 (8.0%) | 0.781 |
| Proven leak only | 44 (3.2%) | 57 (3.3%) | 6 (3.2%) | 107 (3.2%) | 0.99 |

Analysis used chi-square for categorical variables.

Table S4. Rate of complications stratified by manual stapler diameter size

| **Diameter size (mm)** | **<28**  **(N=33)** | **28**  **(N=671)** | **29**  **(N=1,509)** | **30/31**  **(N=766)** | **>31**  **(N=326)** | **Total**  **(N=3,305)** | **P value** |
| --- | --- | --- | --- | --- | --- | --- | --- |
| Proven leak and/or collection | 2 (6.1%) | 57 (8.5%) | 109 (7.2%) | 61 (8.0%) | 35 (10.7%) | 264 (8.0%) | 0.29 |
| Proven leak only | 0 (0.0%) | 23 (3.4%) | 46 (3.0%) | 24 (3.1%) | 14 (4.3%) | 107 (3.2%) | 0.64 |
| Unplanned ICU stay | 0 (0.0%) | 11 (1.6%) | 38 (2.5%) | 17 (2.2%) | 3 (0.9%) | 69 (2.1%) | 0.29 |
| **Pooled analysis** | **Diameter size ≤29 mm** | | | **Diameter size >29 mm** | | **Total** | **P value** |
| Proven leak and/or collection | 169 (7.6%) | | | 96 (8.8%) | | 264 (8.0%) | 0.23 |
| Proven leak only | 69 (3.1%) | | | 38 (3.5%) | | 107 (3.2%) | 0.58 |

Abbreviations: ICU, intensive care unit.

Table S5. Rate of complications stratified by geographic region

|  | **Non-EU (N=228)** | **Eastern Europe (N=369)** | **Northern Europe (N=855)** | **Southern Europe (N=1,290)** | **Western Europe (N=499)** | **Total (N=3,241)** | **P value** |
| --- | --- | --- | --- | --- | --- | --- | --- |
| Proven leak and/or collection | 10 (4.4%) | 32 (8.7%) | 79 (9.2%) | 97 (7.5%) | 40 (8.0%) | 258 (8.0%) | 0.166 |
| Proven leak only | 3 (1.3%) | 9 (2.4%) | 27 (3.2%) | 47 (3.6%) | 18 (3.6%) | 104 (3.2%) | 0.36 |

Abbreviations: EU, European Union.

Table S6. Rate of complications stratified by surgeon in charge

|  | **Trainee**  **(N=253)** | **Consultant**  **(N=3,049)** | **Missing**  **(N=3)** | **Total (N=3,305)** | **P value** |
| --- | --- | --- | --- | --- | --- |
| Proven leak and/or collection | 18 (7.1%) | 246 (8.1%) | 0 | 264 (8.0%) | 0.760 |
| Proven leak only | 8 (3.2%) | 99 (3.2%) | 0 | 107 (3.2%) | 0.95 |

**Detailed list of collaborators and contributions**

Study design, interpretation and writing group: Tong C, Jamous N, Schmitz N-D, Szwarcensztein K, Morton DG, Pinkney TD (chair). Morton DG and Pinkney TD have received funding from Johnson & Johnson. Tong C, Jamous N, Schmitz N-D, Szwarcensztein K are employees of Johnson & Johnson. Pinkney TD had full access to all the data in the study and takes responsibility for the integrity of the data and the accuracy of the data analysis.

ESCP cohort studies and audits committee: El-Hussuna A (2017 ESCP Audit Lead), Battersby N, Bhangu A, Blackwell S, Buchs N, Chaudhri S, Dardanov D, Dulskas A, El-Hussuna A, Frasson M, Gallo G, Glasbey J, Keatley J, Kelly M, Knowles C, Li YE, McCourt V, Minaya-Bravo A, Neary P, Negoi I, Nepogodiev D, Pata F, Pellino G, Poskus T, Sanchez-Guillen L, Singh B, Sivrikoz E, van Ramshorst G, Zmora O, Pinkney TD (chair).

Statistical analysis and data management: Perry R, Magill EL, Keatley J, Tong C.

Collaborators: Ahmed SE, Abdalkoddus M, Abelevich A, Abraham S, Abraham-Nordling M, Achkasov SI, Adamina M, Agalar C, Agalar F, Agarwal T, Agcaoglu O, Agresta F, Ahmad G, Ainkov A, Aiupov R, Aledo VS, Aleksic A, Aleotti F, Alias D, Allison AS, Alonso A, Alonso S, Alós R, Altinel Y, Alvarez-Gallego M, Amorim E, Anania G, Andreev PS, Andrejevic P, Andriola V, Antonacci N, Antos F, Anwer M, Aonzo P, Arenal JJ, Arencibia B, Argeny S, Arnold SJ, Arolfo S, Artioukh DY, Ashraf MA, Aslam MI, Asteria CR, Atif M, Avital S, Bacchion M, Bach SM, Balestri R, Balfour A, Balik E, Baloyiannis I, Banipal GS, Baral JEM, Barišić B, Bartella I, Barugola G, Bass GA, Bedford MR, Bedzhanyan A, Belli A, Beltrán de Heredia J, Bemelman WA, Benčurik V, Benevento A, Bergkvist DJ, Bernal-Sprekelsen JC, Besznyák I, Bettencourt V, Beveridge A J, Bhan C, Bilali S, Bilali V, Binboga E, Bintintan V, Birindelli A, Birsan T, Blanco-Antona F, Blom RLGM, Boerma EG, Bogdan M, Boland MZ, Bondeven P, Bondurri A, Broadhurst J, Brown SA, Buccianti P, Buchs NC, Buchwald P, Bugra D, Bursics A, Burton HLE, Buskens CJ, Bustamante Recuenco C, Cagigas-Fernandez C, Calero-Lillo A, Calu V, Camps I, Canda AE, Canning L, Cantafio S, Carpelan A, Carrillo Lopez MJ, Carvas JM, Carvello M, Castellvi J, Castillo J, Castillo-Diego J, Cavenaile V, Cayetano Paniagua L, Ceccotti AA, Cervera-Aldama J, Chabok A, Chandrasinghe PC, Chandratreya N, Chaudhri SS, Chaudhry ZU, Chirletti P, Chi-Yong Ngu J, Chouliaras C, Chowdhary M, Chowdri NA, Christiano AB, Christiansen P, Citores MA, Ciubotaru C, Ciuce C, Clemente N, Clerc D, Codina-Cazador A, Colak E, Colao García L, Coletta D, Colombo F, Connelly TM, Cornaglia S, Corte Real J, Costa Pereira J, Costa S, Cotte E, Courtney ED, Coveney AP, Crapa P, Cristian DA, Cuadrado M, Cuinas K, Cuk MV, Cuk VV, Cunha MF, Curinga R, Curtis N, Dainius E, d'Alessandro A, Dalton RSJ, Daniels IR, Dardanov D, Dauser B, Davydova O, De Andrés-Asenjo B, de Graaf EJR, De la Portilla F, de Lacy FB, De Laspra ECD, Defoort B, Dehli T, Del Prete L, Delrio P, Demirbas S, Demirkiran A, Den Boer FC, Di Saverio S, Diego A, Dieguez B, Diez-Alonso M, Dimitrijevic I, Dimitrios B, Dimitriou N, Dindelegan G, Dindyal S, Domingos H, Doornebosch PG, Dorot S, Draga M, Drami I, Dulskas A, Dzulkarnaen Zakaria A, Echazarreta-Gallego E, Edden Y, Egenvall M, Eismontas V, El Nakeeb A, El Sorogy M, Elfike H, Elgeidie A, El-Hussuna A, Elía Guedea M, Ellul S, El-Masry S, Elmore U, Emile SH, Enciu O, Enriquez-Navascues JM, Epstein JC, Escolà Ripoll D, Espina B, Espin-Basany E, Estévez Diz AM, Evans MD, Farina PA, Fatayer, Feliu F, Feo C, Feo CV, Fernando J, Feroci F, Ferreira L, Feryn T, Flor-Lorente B, Forero-Torres A, Francis N, Frasson M, Freund MR, Fróis Borges M, Frontali A, Gallardo AB, Galleano R, Gallo G, Garcia D, García Flórez LJ, García Marín JA, García Septiem J, Garcia-Cabrera AM, García-González JM, Garcia-Granero E, Garipov M, Gefen R, Gennadiy P, Gerkis S, Germain A, Germanos S, Gianotti L, Gil Santos M, Gingert C, Glehen O, Golda T, Gómez Ruiz M, Gonçalves D, González, JS, Grainger J, Grama F, Grant C, Griniatsos J, Grolich T, Grosek J, Guevara-Martínez J, Gulcu B, Gupta, SK, Gurjar SV, Haapaniemi S, Hamad Y, Hamid M, Hardt J, Harries RL, Harris GJC, Harsanyi L, Hayes J, Hendriks ER, Herbst F, Hermann N, Heuberger A, Hompes R, Hrora A, Hübner M, Huhtinen H, Hunt L, Hyöty M, Ibañez N, Ignjatovic D, Ilkanich A, Inama M, Infantino MS, Iqbal MR, Isik A, Isik O, Ismaiel M, Ivanovich SO, Jadhav V, Jajtner D, Jiménez Carneros V, Jimenez-Rodriguez RM, Jotautas V, Jukka K, Juloski J, Jung B, Kara Y, Karabacak U, Karachun A, Karagul S, Kassai M, Katorkin Sergei E, Katsaounis D, Katsoulis IE, Kelly ME, Kenjić B, Keogh-Bootland S, Khasan D, Khazov A, Kho SH, Khrykov GN, Kivelä AJ, Kjaer MD, Knight JS, Kocián P, Koëter T, Konsten JLM, Korček J, Korkolis D, Korsgen S, Kostić IS, Krarup PM, Krastev P, Krdzic I, Kreisler Moreno E, Krivokapic Z, Krones CJ, Kršul D, Kumar Kaul N, La Torre F, Lahodzich N, Lai CW, Laina JLB, Lakkis Z, Lamas S, Lange CP, Lauretta A, Lee KA, Lefèvre J, Lehtonen T, Leo CA, Leong KJ, Lepistö A, Licari L, Lizdenis P, Loftås P, Longhi M, Lopez-Dominguez J, López-Fernández J, Lovén H, Lozoya Trujillo R, Lunin R, Luzzi AP, Lydrup ML, Lykke J, Maderuelo-Garcia VM, Madsboell T, Madsen AH, Maffioli A, Majbar MA, Makhmudov A, Makhmudov D, Malik KI, Malik SS, Mamedli ZZ, Manatakis DK, Mankotia R, Maria J, Mariani NM, Marimuthu K, Marinello F, Marino F, Marom G, Maroni N, Maroulis I, Marsanic P, Marsman HA, Martí-Gallostra M, Martin ST, Martinez Alegre J, Martinez Manzano A, Martins R, Maslyankov S, McArdle K, McArthur DR, McFaul C, McWhirter D, Mege D, Mehraj A, Metwally MZ, Metwally IH, Millan M, Miller AS, Minaya-Bravo A, Mingoli A, Minguez Ruiz G, Minusa C, Mirshekar-Syahkal B, Mistrangelo M, Mogoanta SS, Mohamed I, Möller PH, Möller T, Molteni M, Mompart S, Monami B, Mondragon-Pritchard M, Moniz-Pereira Pedro, Montesdeoca Cabrera D, Morais M, Moran BJ, Moretto G, Morino M, Moscovici A, Muench S, Mukhtar H, Muller P, Muñoz-Duyos A, Muratore A, Muriel P, Myrelid P, Nachtergaele M, Nadav H, Nastos K, Navarro-Sánchez A, Negoi I, Nesbakken A, Nestler G, Nicholls J, Nicol D, Nikberg M, Nobre JMS, Nonner J, Norčič G, Norderval S, Norwood MGA, Nygren J, O’Brien JW, O’Connell PR, O'Kelly J, Okkabaz N, Oliveira-Cunha M, Omar GEEI, Onody P, Opocher E, Orhalmi J, Orts-Micó FJ, Ozbalci GS, Ozgen U, Ozkan BB, Ozturk E, Pace K, Padín MH, Pandey SB, Pando JA, Papaconstantinou I, Papadopoulos A, Papadopoulos G, Papp G, Paraskakis S, Parc Y, Parra Baños P, Parray FQ, Parvuletu R, Pascariello A, Pascual Migueláñez I, Pata F, Patel H, Patel PK, Paterson HM, Patrón Uriburu JC, Pattacini GC, Pavlov V, Pcolkins A, Pellicer-Franco EM, Peña Ros E, Pérez HD, Petkov P, Picarella P, Pikarsky AJ, Pisani Ceretti A, Platt E, Pletinckx P, Podda M, Popov D, Poskus E, Poskus T, Prats MC, Pravosudov I, Primo-Romaguera V, Prochazka V, Pros Ribas, I, Proud D, Psaila J, Pullig F, Qureshi Jinnah MS, Rachadell Montero J, Radovanovic D, Radovanovic Z, Rahman MM, Rainho R, Rama N, Ramos D, Ramsanahie, A, Rantala A, Rasulov A, Rautio T, Raymond T, Raza A, Reddy A, Refky B, Regusci L, Reissman P, Rems M, Reyes-Diaz ML, Riccardo R, Richiteanu G, Richter F, Rios A, Ris F, Rodriguez FL, Rodriguez Garcia P, Rojo Lopez JA, Romaniszyn M, Romano GM, Romero AS, Romero-Simó M, Roshan Lal A, Rossi B, Ruano Poblador A, Rubbini  M, Rubio-Perez I, Ruiz H, Rullier E, Ryska O, Sabia D, Sacchi M, Saffaf N, Sakr A, Saladzinskas Z, Sales I, Salomon M, Salvans S, Samalavicius NE, Sammarco G, Sampietro GM, Samsonov D, Sanchez-Garcia JL, Sánchez-Guillén L, Sanchiz E, Šantak G, Santos Torres J, Saraceno F, Sarici IS, Sarmah PB, Savino G, Scabini S, Schafmayer C, Schiltz B, Schofield A, Scurtu R, Segalini E, Segelman J, Segura Sampedro JJ, Seicean R, Sekulic A, Selwyn D, Serrano Paz P, Shabbir J, Shaikh IA, Shalaby M, Sharma A, Shukla A, Shussman N, Siddiqui ZA, Siironen P, Sileri P, Silva-Vaz P, Simoes JF, Sinan H, Singh B, Sivins A, Skroubis G, Skrovina M, Skull AJ, Slavchev M, Slavin M, Slesser AAP, Smart CJ, Smart NJ, Smedh K, Smolarek S, Sokolov M, Sotona O, Spacca D, Spinelli A, Stanojevic G, Stearns A, Stefan S, Stift A, Stijns J, Stoyanov V, Straarup D, Strouhal R, Stubbs BM, Suero Rodríguez C, Sungurtekin U, Svagzdys S, Svastics I, Syk I, Tabares MJM, Tamelis A, Tamhane RG, Tamini N, Tamosiunas A, Tan SA, Tanis PJ, Tate SJ, Tercioti Junior V, Terzi C, Testa V, Thaha MA, Tham JC, Thavanesan N, Theodore JE, Tinoco C, Todorovic M, Tomazic A, Tomulescu V, Tonini V, Toorenvliet BR, Torkington J, Torrance A, Toscano MJ, Tóth I, Trampus S, Travaglio E, Trostchanky I, Truan N, Tulchinsky H, Turrado-Rodriguez V, Tutino R, Tzivanakis A, Tzovaras GA, Unger LW, Vaccari S, Vaizey CJ, Valero-Navarro G, Valverde Nuñez I, Van Belle K, Van Belle K, van den Berg I, van Geloven AAW, Van Loon YT, van Steensel L, Varcada M, Vardanyan AV, Varpe P, Velchuru VR, Vencius J, Venskutonis D, Vermaas M, Vertruyen M, Vicente-Ruiz M, Vignali A, Vigorita V, Vila Tura M, Vimalachandran D, Vincenti L, Viso L, Visschers RGJ, Voronin YS, Walega P, Wan Zainira WZ, Wang JH, Wang X, Wani R, Warusavitarne J, Warwick A, Wasserberg N, Weiss DJ, Westerduin E, Wheat JR, White I, Williams G, Williams GL, Wilson TR, Wilson JM, Winter D, Wolthuis AM, Wong MPK, Worsøe J, Xynos E, Yahia S, Yamamoto T, Yanishev A, Zaidi Z, Zairul Azwan MA, Zaman S, Zaránd A, Zarco A , Zawadzki M, Zelic M, Žeromskas P, Zilvetti M, Zmora O
